# Staphylococcal Virulence Factors on the Skin of Atopic Dermatitis Patients

**DOI:** 10.1128/mSphere.00616-19

**Published:** 2019-12-11

**Authors:** Mary C. Moran, Michael P. Cahill, Matthew G. Brewer, Takeshi Yoshida, Sara Knowlden, Nelissa Perez-Nazario, Patrick M. Schlievert, Lisa A. Beck

**Affiliations:** a Department of Microbiology and Immunology, University of Rochester Medical Center, Rochester, New York, USA; b Department of Dermatology, University of Rochester Medical Center, Rochester, New York, USA; c Department of Microbiology and Immunology, University of Iowa, Iowa City, Iowa, USA; University of Nebraska Medical Center

**Keywords:** *Staphylococcus aureus*, atopic dermatitis, infections, skin, superantigens

## Abstract

Staphylococcus aureus is the leading cause of skin and soft tissue infections, bacteremia, infective endocarditis, osteoarticular, pleuropulmonary, and device-related infections. Virulence factors secreted by S. aureus, including superantigens and cytotoxins, play significant roles in driving disease. The ability to identify virulence factors present at the site of infection will be an important tool in better identifying and understanding how specific virulence factors contribute to disease. Previously, virulence factor production has been determined by culturing S. aureus isolates and detecting the mRNA of specific virulence factors. We demonstrated for the first time that virulence factors can be directly detected at the protein level from human samples, removing the need to first culture isolated bacteria. Superantigens and cytotoxins were detected and quantified with a Western dot blot assay by using reconstituted skin swabs obtained from patients with atopic dermatitis. This methodology will significantly enhance our ability to investigate the complex host-microbe environment and the effects various therapies have on virulence factor production. Overall, the ability to directly quantify virulence factors present at the site of infection or colonization will enhance our understanding of S. aureus-related diseases and help identify optimal treatments.

**IMPORTANCE** For the first time, we show that secreted staphylococcal virulence factors can be quantified at the protein level directly from skin swabs obtained from the skin of atopic dermatitis patients. This technique eliminates the need to culture Staphylococcus aureus and then test the strain’s potential to produce secreted virulence factors. Our methodology shows that secreted virulence factors are present on the skin of atopic patients and provides a more accurate means of evaluating the physiological impact of S. aureus in inflammatory diseases such as atopic dermatitis.

## Abstract (precis)

For the first time, we show that secreted staphylococcal virulence factors can be quantified at the protein level directly from skin swabs obtained from the skin of atopic dermatitis patients. This technique eliminates the need to culture Staphylococcus aureus and then test the strain’s potential to produce secreted virulence factors. Our methodology shows that secreted virulence factors are present on the skin of atopic patients and provides a more accurate means of evaluating the physiological impact of S. aureus in inflammatory diseases such as atopic dermatitis.

## INTRODUCTION

Staphylococcus aureus infections have a significant impact on human health and health care costs (1). In the United States alone, there are more than 500,000 surgical site skin infections yearly and countless cases of other soft tissue infections. These skin infections cost the U.S. health care system billions of dollars yearly (2). The increasing prevalence of antibiotic-resistant S. aureus strains further complicates the ability to treat S. aureus infections and poses a significant threat to public health. In 2007, the Centers for Disease Control and Prevention (CDC) stated that S. aureus is the most significant cause of serious infections (3).

S. aureus causes skin infections in patients with diabetes, sometimes resulting in foot ulcers, limb amputations, and death. As many as 30 million S. aureus diabetic infections are reported yearly in the United States (4, 5). Patients with atopic dermatitis or eczema are another population affected by both colonization and infections with S. aureus. This disease is characterized by skin barrier disruption, type 2 immunity, enhanced IgE production, and an altered microbiota with S. aureus dominance (6–11). S. aureus colonization and infections are thought to contribute to the severity and persistence of this disease (12). Atopic dermatitis is the most common inflammatory skin disorder and is estimated by the World Health Organization to impact 230 million people worldwide.

S. aureus is thought to drive inflammation at least in part by the production of a number of virulence factors that may, among other things, disrupt epithelial barriers, promote immune evasion, and enhance inflammation. S. aureus produces both bacterial cell surface and secreted virulence factors. Many atopic dermatitis studies have implicated a role for these secreted virulence factors in both causing and maintaining the condition (13–21).

Major classes of secreted virulence factors include superantigens, cytotoxins, proteases, and lipase (22, 23). Superantigens include toxic shock syndrome toxin-1 (TSST-1), staphylococcal enterotoxins (SEs), and staphylococcal enterotoxin-like toxins (SEls) (24). Superantigens are commonly secreted by S. aureus; however, superantigen genes have rarely been detected in select coagulase-negative staphylococci, including Staphylococcus epidermidis, Staphylococcus warneri, and Staphylococcus haemolyticus (25, 26). Studies suggest that one or more superantigens and cytotoxins must be present for S. aureus to colonize and cause human infections (27). Superantigens play critical roles in the development and progression of disease, such as toxic shock syndrome (TSS), staphylococcal food poisoning, pneumonia, infective endocarditis, atopic dermatitis, and type II diabetes. For example, 100% of menstrual TSS is caused by TSST-1, and staphylococcal food poisoning is caused by SEs A to E (28–30). There are at least five major cytotoxins; α, β, γ (and related leukocidins), δ, ε, and the large family of phenol-soluble modulins (PSMs). All of these proteins likely contribute in unique ways to a number of human diseases.

To date, no one has shown conclusively that secreted virulence factors are produced at sites of infection. Identification of such virulence factors at infection sites would greatly enhance our understanding of how S. aureus and specific virulence factors promote disease progression and persistence.

Previously, secreted virulence factors were indirectly detected by culturing S. aureus isolates from patients and analyzing the culture media for superantigen and cytotoxin mRNA or proteins (22). This approach of removing bacteria from the skin environment, where interactions with other microbes and host cells are likely influencing virulence gene expression and S. aureus growth characteristics, may actually yield misleading information. Here, we show that secreted staphylococcal virulence factors (superantigens and cytotoxins) can be quantified directly, without culture, at the protein level from swabs obtained from atopic dermatitis patients and healthy control nonpurulent skin. For the first time, we demonstrate the presence of these virulence factors at sites of skin colonization in both diseased and healthy human subjects.

## RESULTS AND DISCUSSION

**FIG 1:**
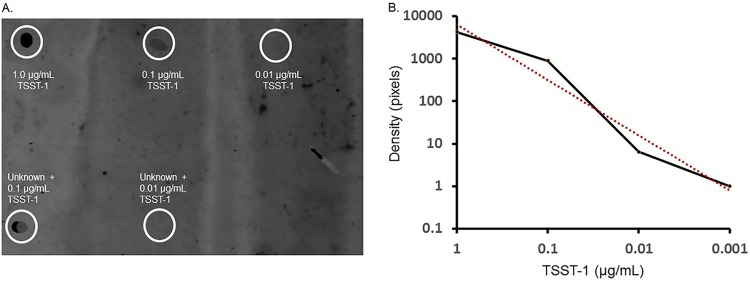
Quantitative Western dot blot and standard curve of TSST-1. (A) Known concentrations of TSST-1 were spotted on PVDF membranes (top row, left to right: 1.0, 0.1, and 0.01 μg/ml; second row, not circled due to lack of reactivity, left to right: 0.001, 0.0001, and 0 μg/ml). Skin swabs were spiked with 0.1 and 0.01 μg/ml TSST-1 (bottom left, middle). (B) A standard curve of TSST-1 was made by measuring the densities with ImageJ; 0.001 μg/ml TSST-1 was undetectable. A line of best fit is in red.

We assumed that the amounts of superantigens and cytotoxins *in vivo* on human skin may be low, possibly in the microgram to nanogram per milliliter amounts, if present at all. Thus, we needed to develop a sensitive method for direct quantification of the proteins. The Schlievert laboratory has specific antibodies to nearly all secreted virulence factors. This allowed us to develop a Western dot blot for direct detection. Figure 1A shows an example of a Western dot blot for TSST-1 at six known concentrations (1.0, 0.1, 0.01, 0.001, 0.0001, and 0 μg/ml) as well as two spiked unknowns in which a 2 -cm by 2-cm area of skin was swabbed and the swab was then dipped in 0.1 or 0.01 μg/ml of TSST-1. This was to test if skin interferes with the detection of TSST-1. ImageJ was used to calculate the densities of each blot and create a standard curve, as shown in Fig. 1B. Using this method, we were able to determine that the skin swab does not interfere with the detection of TSST-1, as the density of the unknowns was 0.12 ± 0.02 μg/ml when spiked with 0.1 μg/ml of TSST-1 and 0.02 ± 0.01 μg/ml when spiked with 0.01 μg/ml of TSST-1.

**FIG 2:**
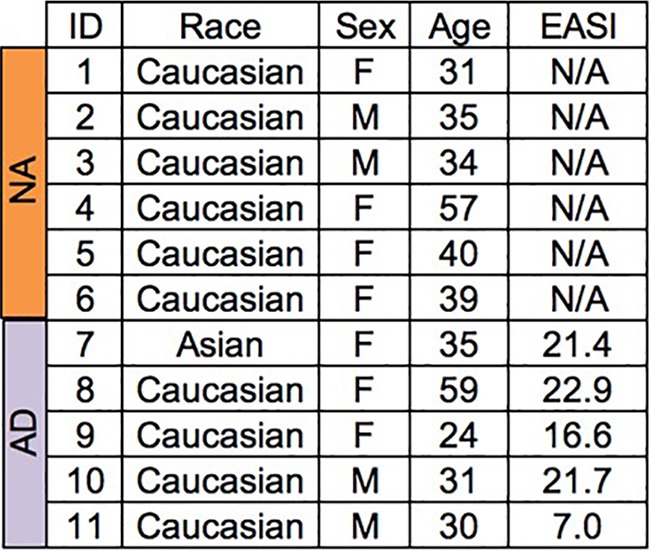
Patient demographics. Mild AD, EASI of 1 to 7; moderate AD, EASI of 7.1 to 21; severe AD, EASI of 21.1 to 50; NA, nonatopic; AD, atopic dermatitis; EASI, eczema area and severity index.

To determine if staphylococcal virulence factors could be detected at the protein level directly from skin swabs, patients with atopic dermatitis were enrolled in a small pilot study in which skin swabs were collected from lesional and nonlesional sites (Fig. 2). Patients with atopic dermatitis were selected as the population of interest because upwards of 90% of patients are colonized on their skin with S. aureus compared to less than 5% of healthy individuals (31, 32). Furthermore, previous reports in which S. aureus isolates were cultured and superantigen mRNA was measured in culture media have shown that disease severity correlates with the presence of superantigen-producing S. aureus colonizing the skin (33). We hypothesized that due to the high prevalence of S. aureus colonization in this population, as well as the superantigen-producing ability of atopic dermatitis S. aureus isolates, superantigens and cytotoxins would be detectable at the protein level in reconstituted skin swabs from these patients.

**FIG 3:**
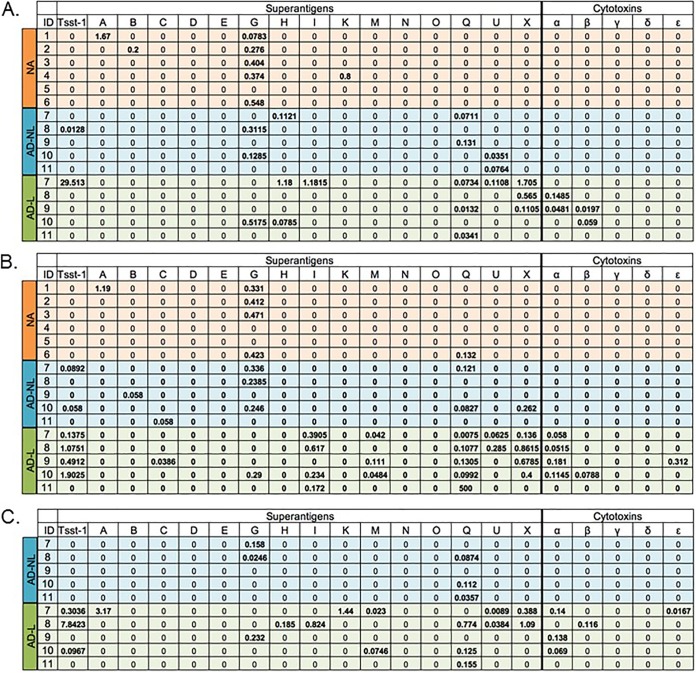
Quantification of superantigens and cytotoxins detected in skin swabs. Superantigens and cytotoxins were detected and quantified from reconstituted skin swabs by Western dot blot. Units are micrograms per milliliter. Swabs were collected at initial visit (A), 1 week post bleach bath intervention (B), and 6 weeks post bleach bath intervention (C). One swab was collected from nonatopic (NA) patients. Two nonlesional and two lesional swabs were collected from atopic dermatitis patients (AD-NL, AD-L) at the initial and 1-week visits. Concentrations of the two swabs were averaged.

Skin swabs, collected from atopic dermatitis patients and healthy controls, were reconstituted in phosphate-buffered saline, and Western dot blots using rabbit antibodies specific to superantigens and cytotoxins were used to detect the presence of the virulence factors. The concentration of each virulence factor present in the swab was determined using ImageJ by comparing the densities of our samples to the densities of control virulence factors in which the concentration used in the Western dot blot assay was known. Using this methodology, we demonstrated for the first time that superantigens and cytotoxins can be measured at the protein level directly from skin swabs (Fig. 3). Interestingly, cytotoxins were only detected in swabs collected from the lesional sites of atopic dermatitis patients (Fig. 3). This is consistent with the current thinking that cytotoxins are critical for causing skin infections (27). A larger sample of swabs from nonlesional and lesional sites of atopic dermatitis patients and healthy individuals would be needed to compare the superantigen and cytotoxin profiles across the disease states. This study demonstrates that the skin swab and Western dot blot methodology are capable of directly quantifying these virulence factors and provides the necessary system for comparative studies in the future.

**FIG 4:**
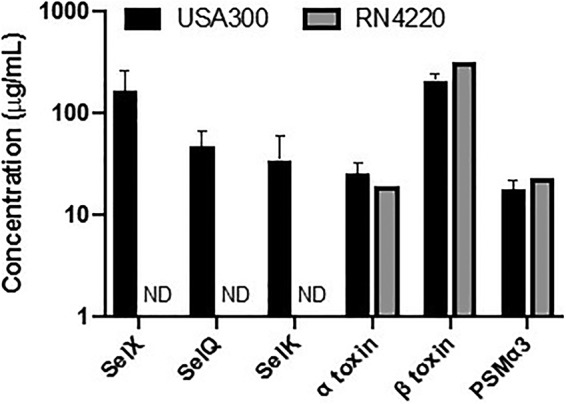
Quantification of USA300 and RN4220 supernatant fluids. Overnight cultures of the S. aureus strains USA300 and RN4220 were grown in tryptic soy broth. Culture media were filtered twice by 0.2-μm filters. Superantigens and cytotoxins were detected in the supernatant fluids using our developed quantitative Western dot blot.

We further verified the ability to measure superantigens and cytotoxins at the protein level by measuring the concentrations of these virulence factors in the filtered culturing media of the S. aureus strains USA300 (FRP3757; a derivative of the LAC strain) and RN4220. The virulence factors produced by these strains have been well documented in the literature and therefore served as good controls to validate our methodology. The USA300 lineage of S. aureus is commonly associated with skin and soft tissue infections and has been reported to produce SElK, SElQ, SElX, α-toxin, and β-toxin (34). The RN4220 strain of S. aureus is a derivative of the NCTC8325 isolate and does not produce any superantigens but does produce α-toxin and β-toxin (35). The superantigen and cytotoxins detected using the quantitative Western dot blot in the culturing media were in agreement with the previously reported expression profiles for these two commonly utilized strains, further validating that the quantitative Western immunoblot can be used to detect superantigens and cytotoxins at the protein level (Fig. 4).

The ability to directly, and at the protein level, measure staphylococcal virulence factors present on the skin is a significant methodologic breakthrough. These methods, in which the virulence factor profile can be accurately quantified in the environment of interest, provide a more accurate snapshot of virulence factor production at a specific point in time, specific anatomical site, and as a consequence of the specific micro- (e.g., microbial flora) and macroenvironmental influences. Both the host and other microbes release factors that, in addition to the quorum sensing system of S. aureus itself, alter virulence factor production by S. aureus. These signals are lost when employing the previously used *in vitro* methods of culturing S. aureus and quantifying virulence factor mRNA after *in vitro* culture.

This assay has vast potential for significantly improving our understanding of how staphylococcal virulence factors may drive diseases that are associated with or caused by S. aureus colonization/infection. We propose a number of studies in which this methodology can be applied to study the relationship between S. aureus virulence factors and atopic dermatitis. This assay makes it possible to quantify changes in the superantigen profile that arise in response to a targeted intervention in atopic dermatitis patients using a longitudinal study design. With high frequency of sampling, we may learn how disease severity affects S. aureus virulence factor expression patterns and vice versa. Another potential use of this new methodology might be to avoid steroid treatment in atopic dermatitis patients who have superantigens linked to the development of a steroid resistance state and, in doing so, adopt a more personalized medicine approach to the treatment of this disease (33, 36). These assays are opportunities to better understand how specific S. aureus virulence factors may impact persistence of S. aureus colonization as well as susceptibility to a number of cutaneous viruses (e.g., herpes simplex virus, vaccinia virus, and human papillomavirus), which is a characteristic of the atopic dermatitis population (6, 37, 38). This methodology opens new avenues to better understand the biological consequences of S. aureus virulence factors on a number of diseases, including atopic dermatitis, which will likely help us identify the best way to manage both their comorbidities and the disease itself.

## MATERIALS AND METHODS

### Human skin swab sample collection.

Skin swabs were collected from six nonatopic (NA) and five atopic dermatitis (AD) adult patients. A 3- cm by 3-cm area of skin was swabbed from each volunteer using the Catch-All Sample Collections swab (Epicentre, Madison, WI), which was premoistened with nonbacteriostatic sterile saline and stored in 2-ml Eppendorf Safe-Lock tubes (Biopur) at −80°C. Skin swabs were collected from AD patients at both lesional (AD-L) and nonlesional (AD-NL) sites at three time points: baseline (T1), 1 week into bleach baths (T2), and 6 weeks into bleach baths (T3). Swabs were collected from NA individuals at T1 and T2. A single swab at T1 and T2 was collected from NA individuals whereas AD patients had two consecutive swabs at lesional and nonlesional sites taken at T1 ant T2 and only one swab collected at T3. All AD patients were S. aureus positive by culturing, and culture positivity did not significantly change in AD patients as a consequence of biweekly bleach bath treatments (data not shown). NA patients were all S. aureus negative by culturing. Swabs were reconstituted in phosphate-buffered saline (PBS), and superantigens and cytotoxins were detected by a quantitative Western immunoblot assay.

### S. aureus control strain culturing.

The S. aureus strains USA300 (FRP3757) and RN4220 were grown overnight shaking at 37°C in tryptic soy broth. Culture media were then centrifuged at 7,000 × *g* for 10 min and filtered through 0.2-μm filters twice to remove bacteria.

### Quantitative Western dot blot assay.

Superantigens and cytotoxins in participant samples were detected by a quantitative Western dot blot assay. Rabbits were first hyperimmunized three times, every other week with highly purified superantigens and cytotoxins. The animals were then tested for antibodies to the corresponding superantigen or cytotoxin after drawing a small sample of blood from the marginal ear vein. Serum from the animals reacted to the same extent with purified superantigens and cytotoxin (50 μg/ml) when tested by double immunodiffusion. This suggested that the antibody titers against each superantigen and cytotoxin were similar and likely to react similarly in Western dot blots. This was the case, as standard curves were similar for reactivity of hyperimmune serum with immunizing superantigen or cytotoxin.

To establish a standard curve and to validate the new procedure, various TSST-1 concentrations (1 μg/ml to 10^−4^ μg/ml) were spotted in 5-μl volumes onto polyvinylidene (PVDF) membranes (Bio-Rad Laboratories, Hercules, CA). TSST-1 samples were first suspended in sterile water containing 0.1% bovine serum to eliminate background readings. Additionally, a standard cotton swab containing 0.1 ml of phosphate-buffered saline (PBS) was rolled in two directions across a 2-cm by 2-cm square of forearm skin from a healthy volunteer. The swab was immersed in a standard solution of TSST-1 (0.1 or 0.01 μg/ml), which was then centrifuged at 14,000 × *g* for 5 min. The clarified supernate (5 μl) was spotted onto the same PVDF membrane. Then, all samples were dried. The PVDF membrane was then developed as a Western dot blot, first with blocking with 5% milk and then developed with hyperimmune rabbit polyclonal antibodies raised against TSST-1 and then with LI-COR (Lincoln, NE) IRDye 680LT-labeled goat antibodies against rabbit antibodies. The samples were scanned with a LI-COR Odyssey CLx. Spot densities were determined with use of NIH ImageJ. Participant samples were developed similarly.
